# Comparative Context of Hard‐Tissue Sexual Dimorphism in Early Hominins: Implications for Alpha Taxonomy

**DOI:** 10.1002/evan.22052

**Published:** 2025-01-02

**Authors:** Katharine L. Balolia, Bernard Wood

**Affiliations:** ^1^ School of Archaeology and Anthropology Australian National University Canberra Australia; ^2^ Department of Anthropology Durham University Durham UK; ^3^ Center for the Advanced Study of Human Paleobiology George Washington University Washington DC USA

**Keywords:** cranium, growth, *Paranthropus*, primate, sex differences, skeleton

## Abstract

Sexual dimorphism is one of the main factors confounding attempts to generate sound alpha taxonomic hypotheses in the early hominin fossil record. To better understand how between‐sex variation may confound alpha taxonomic assessments, we consider some of the factors that drive hard‐tissue sexual dimorphism in extant primates. We review the socioecological correlates of body size sexual dimorphism, how sexual selection may be associated with craniofacial sexual dimorphism in the context of visual signaling, and how sex‐specific patterns of growth and development in primates contribute to intra‐specific variation. To illustrate how variation associated with inferred sexual dimorphism has the potential to confound alpha taxonomic assessments in early hominins, we focus on its impact on our understanding of a single taxon, *Paranthropus boisei*. We suggest that regions of the skeleton likely to be influenced by sexual selection should be avoided when generating alpha taxonomic hypotheses.

## 1 Introduction

Sexual dimorphism influences how we interpret the hominin fossil record in two main ways. First, it confounds attempts to generate reliable alpha taxonomic hypotheses because high levels of size and shape craniofacial sexual dimorphism could be mistaken for inter‐specific variation, resulting in the overestimation of taxic diversity (e.g. [1, 2, 3, 4, 5]). The second way sexual dimorphism influences the interpretation of the hominin fossil record is that once sound alpha taxonomic hypotheses have been generated, reference models based on sexual dimorphism in extant primates are used to make behavioral inferences about early hominin taxa (e.g., [6, 7]).

Our primary aim in writing this article is to provide a comparative context for the task of assessing the influence of sexual dimorphism on attempts to generate reliable alpha taxonomic hypotheses. A comprehensive review of the biological basis of sexual dimorphism, its expression in extant primates, and its possible expression in the hominin fossil record, is much too large an undertaking for a review of this scale. Instead, we focus on the different research interests that led each of us to our shared interest in craniofacial sexual dimorphism. One of us (B.W.) investigated sexual dimorphism because he needed to understand more about the potential contribution of sexual dimorphism to intra‐specific variation when he was charged with working out how many early hominin taxa were being sampled at Koobi Fora [8]. This perspective is very different from K.B.'s research interest, which explores ways in which sexual selection may have driven aspects of craniofacial sexual dimorphism in extant and fossil hominoids (e.g., [9, 10, 11]).

Appreciating the nature and extent of the contribution of sexual dimorphism to intra‐specific variation in the early hominin fossil record is essential if we are to generate sound alpha taxonomic hypotheses [12], but showing how sexual dimorphism potentially confounds alpha taxonomy across all of the early hominin fossil record is beyond the scale and scope of this review, so we use one taxon, *Paranthropus boisei*, as an example of how variation due to sexual dimorphism might be manifest in the early hominin fossil record. We also explore the ways sexual dimorphism contributes to intra‐specific craniofacial variation within extant primates to better understand the difference between intra‐ and inter‐species hard‐tissue variation in early hominins. A comprehensive review of what is known, and not known, about sexual dimorphism in the hominin fossil record, is the topic of an upcoming review.

There are several ways that selective pressures associated with sexual selection, which are difficult to ascertain a priori among hominin fossil assemblages, may be causally associated with the expression of sexual dimorphism within a taxon. Evidence from extant primates shows that the influence of sexual dimorphism on hard‐tissue size and shape variation varies across the regions of the skeleton [13], and there may even be differences in the degree of sexual dimorphism within the same region [14]. In this article, we will show that primate species may exhibit high between‐sex shape variation in some skeletal regions, but low between‐sex shape variation in others, associated with sexual or social selection depending on aspects of the socioecology of the species under scrutiny. Identification of regions that vary in response to social or sexual selection among extinct hominin species is an important consideration for evaluating alpha taxonomic hypotheses. In light of our primary aim, which is to understand how the influences of sexual dimorphism may confound attempts to generate reliable alpha taxonomic hypotheses, we consider regions of the skeleton that may have been influenced by sexual selection, but we do not review the underlying basis for canine crown height dimorphism because a reduction in the size of the canine teeth is one of the main criteria used to recognize hominins (e.g., [15]). Understanding sexual dimorphism as a source of intra‐specific variation allows us to better retrodict how sexual size and shape dimorphism contributed to the morphological variation observed in fossil assemblages. We use *P. boisei* [16, 17] as an example of how sexual dimorphism needs to be taken into account when generating alpha taxonomic hypotheses, but we provide a more wide‐ranging review of the fossil evidence in a follow‐up review. We propose that skeletal regions that are likely to vary in response to sexual or social selection among hominins should be avoided when generating alpha taxonomic hypotheses, and we discuss what information we may be missing by being limited to the hard tissue sexual dimorphism that is captured by the hominin fossil record. We appreciate that factors other than sexual dimorphism (e.g., behavior, geography, time, ontogeny, and taphonomy) contribute to intra‐specific variation in the hominin fossil record, but aside from brief reference to some of these variables in our example, we do not consider them further in this review.

## 2 Causes of Sexual Dimorphism

In this section we consider some possible underlying causes of body weight/body size sexual dimorphism among primates, and provide a brief overview of the influence of socioecological factors such as mating system, availability of females and the associated frequency and intensity of inter‐male competition. We also briefly review selective pressures that have the potential to influence craniofacial dimorphism, including whether some craniofacial regions may be under selection as a source of visual signaling, and how sexual dimorphism might relate to sex‐specific patterns of ontogeny and the extension of skeletal growth beyond dental maturity. The purpose of our review of these underlying causes of sexual dimorphism is to better understand how these sources of variation may confound attempts to generate reliable alpha taxonomic hypotheses within the hominin clade. Plavcan [18, 19, 20, 21] and Cassini [22] provide wider‐ranging reviews of the potential causes of sexual dimorphism among primates.

### 2.1 Body Weight Dimorphism

The main way in which sexual dimorphism may influence attempts to generate alpha taxonomic hypotheses among fossil assemblages is through the association between body size dimorphism and skeletal dimorphism among primates [23]. There has been much research investigating the relationship between body weight or body size dimorphism and intra‐sexual competition, through direct observations (such as the nature and frequency of inter‐male competitive encounters), or by looking at aspects of primate sociality, including mating system, social organization or the socionomic or operational sex ratio. Early studies on this topic suggested a relationship between mating system and the degree of body weight sexual dimorphism, with polygynous primates displaying higher levels of body weight dimorphism than monogamous ones, with the heaviest primate species being the most dimorphic [24, 25, 26]. Subsequent research showed that species in which males show intense and frequent intra‐sexual competition and aggression, or the highest ratios of fertile females to reproductively active males in a group (as measured by the operational sex ratio), show increased selection for larger male body weights [20, 27, 28, 29]. Natural selection has also been proposed as a driver of sexual size dimorphism among primates, through differences in the way each sex interacts with their environment, independent of pressures associated with sexual selection [30, 31]. It is known that aspects of primate socioecology, such as group size, social organization and mating system, are influenced by ecological factors such as food availability, which in turn affects the distribution of females and the associated ability of males to monopolize access to females [32]. All of this makes it difficult to tease out the relative effects of selection, both natural and sexual, on the expression of body size sexual dimorphism among primates.

### 2.2 Craniofacial Dimorphism in the Context of Visual Signaling

A less‐explored consideration concerning the expression of sexual dimorphism is whether craniofacial regions undergo selection in the context of visual signaling, which has implications for interpreting the underlying sources of variation among extinct hominin fossil assemblages, especially given that their relatively small canines mean there were likely other ways that early hominins signaled their status. Research indicates there is greater complexity in the secondary sexual adornments of primate males who live in uni‐male/multi‐female social organizations, with evidence that sexual dimorphism in visually conspicuous traits occurs in addition to any influence body mass may have on sexual dimorphism. These adornments include facial flanges in adult male orangutans, bright skin coloration in adult male mandrills, and large bulbous noses in adult male proboscis monkeys (as reviewed by [33]). If the hard tissues underlying these sexually dimorphic soft tissue facial traits also vary, this may be an additional source of sex‐related skeletal variation. For example, in the upper face, browridge morphology among extinct hominins cannot be explained solely through mechanical considerations [34, 35], with the possibility that eyebrow mobility may be associated with social signaling [36]. Similarly, there is evidence that browridge morphology among red colobus monkeys and some hylobatid taxa may vary in response to socioecological factors [10, 37]. With respect to the mid‐face, nasal cavity size and shape among proboscis monkeys is associated with visual and acoustic signaling [11]. Among modern humans and non‐human primates, relatively wide faces are associated with behavioral and personality variables, as well as perceived dominance and aggression [38, 39, 40, 41, 42, 43, 44], but these associations are not universal among species or populations [45].

Some have suggested the sagittal crest may function as a visual signal. Among western lowland gorillas, sagittal hump size in males is associated with the number of females associated with each male in adulthood and the number of successfully‐reared offspring [46, 47]. Similarly, among mountain gorilla males, back breadth and sagittal crest height are positively associated with dominance rank, group tenure length and the number of females per group [48]. In both western lowland gorillas and Bornean orangutans, sagittal crest development coincides with sexual maturity in the males of both species, which is consistent with it being a sexually selected trait [9]. Overall, these findings suggest sexual selection and social signaling may influence sexual dimorphism in some regions of the craniofacial skeleton, resulting in a high degree of intra‐specific variation among these morphological traits. If this is the case, sex‐related variation in these regions could confound attempts to generate reliable alpha taxonomic hypotheses. We discuss this further in Section 4.

### 2.3 Sex Differences in Skeletal Growth and Development

Sex differences in growth and development are another source of variation associated with sexual dimorphism. Most of the research exploring sex‐specific ontogenetic patterns of growth and development in the primate skeleton focuses on the cranium. Researchers have shown there are differences among taxa in how craniofacial sexual dimorphism is attained among primates [49]. Some species, including crab‐eating macaques and red howler monkeys, predominantly attain adult sexual dimorphism through sex differences in rates of growth (rate hyper‐ or hypomorphosis) [50, 51, 52]. Other species, including proboscis monkeys, mantled howler monkeys, tufted capuchin monkeys and collared mangabeys, attain adult sexual dimorphism mainly through sex differences in the timing of growth cessation (bimaturism, or duration hyper‐ or hypomorphosis) [50, 51, 53]. Craniofacial sexual dimorphism among many primate taxa can be attained through a combination of rate and duration hyper‐ or hypomorphosis (e.g., among some African apes, orangutans, rhesus macaques and papionins [54, 55, 56, 57, 58, 59, 60]). Similar levels of sexual size dimorphism can therefore be attained either through sex differences in the rate of growth, sex differences in the duration of growth, or a combination of the two [49, 58]. Sex differences in craniofacial ontogeny among primates are often consistent with the presence of sex differences in patterns of body mass growth, where males of some species may grow for longer than females, or growth may be delayed, followed by an adolescent, or adulthood growth spurt [26, 61, 62, 63, 64, 65, 66]. Studies among gorillas, who manifest high levels of body size dimorphism, suggest there are sex differences in the timing and rate of postcranial and body size growth across taxa and sexes [67, 68].

How sexual dimorphism is attained is associated with aspects of primate socioecology. For example, in some Old World monkey and ape species, males delay growth relative to females. This is followed by an adolescent growth spurt associated with a delay in the emergence of characteristics that signal social maturity, until males are ready to compete with other males [61, 62, 69]. There are also sex differences in the timing of growth cessation associated with the timing of social maturity during early‐ to mid‐adulthood among anthropoids [9, 61, 63, 69, 70].

As noted above, many studies in primates have demonstrated skeletal variation associated with growth and development until the onset of dental maturity, and this source of variation is taken into account when considering potential influences on variation among fossil hominin taxa [54, 71, 72, 73]. However, evidence for skeletal growth and development beyond dental maturity is less well documented, and among some highly sexually‐dimorphic non‐human primate taxa, including gorillas and orangutans, male craniofacial growth continues well beyond dental maturity [9, 70, 74]. Among orangutans, growth can be “indeterminate” [9, 57, 59, 60, 70, 74]. The timing of male growth cessation may be linked with energetic constraints, and the time required for males to attain full body size [9, 57, 59, 60, 70, 75, 76, 77]. In some non‐human primate taxa, females also show growth beyond dental maturity [70, 75].

Evidence among fossil hominins of growth beyond dental maturity [78, 79] suggests that differences in this phenomenon may influence sex‐related intra‐specific variation in early hominins, either based on morphological variation associated with body size dimorphism (reviewed in Section 2.1), selection on specific craniofacial traits associated with visual signaling (reviewed in Section 2.2) or a combination of the two. Growth beyond dental maturity in either sex has the potential to influence the degree of intra‐specific variation present within fossil assemblages, making it difficult for researchers to reliably generate alpha taxonomic hypotheses.

### 2.4 Male and Female Contributions to Sexual Dimorphism

The majority of sexual dimorphism research among primates has focused on the selective pressures that contribute to variation in male morphology, either through sexual selection via male‐male competition or through female mate choice (as reviewed by [80, 81, 82]). For example, as noted above, variation in male body size, canine crown height and facial characteristics has been associated with levels of inter‐male aggression, mating system and social organization ([20, 29, 83]; (as reviewed by [33]). Less well‐studied is whether, and how, selective pressure on female morphology contributes to sexual dimorphism ([84, 85]; as reviewed by [19]). While some of the variation in female body size is associated with correlated response or selection, in association with selection on male body size, there is scope for natural selection to also act on female body size, independent of selective pressures influencing male body size [19, 84]. For example, female resource competition may favor larger female body size because larger females are able to give birth to larger offspring [19, 86], and larger body size may afford further advantages associated with higher dominance rank and increased access to preferred food resources [19, 86, 87]. Conversely, body size can be inversely related to fecundity because smaller individuals are able to reproduce more often [19, 31, 86, 88].

Consistent with findings that female body size is likely under selection because of intra‐sexual competition, some authors have adopted a social selection approach to understanding behavior, including the possibility of intra‐specific competition for resources among males as well as females [89, 90, 91]. Others have adopted approaches that entertain independent selective pressures acting on males and females (e.g., [91, 92, 93]). There is evidence in some primate species that when body size and craniofacial growth in females extends beyond the onset of dental maturity, it may result in a higher dominance rank [63, 70, 75, 76].

In summary, selective pressures on both sexes potentially contribute to sexual dimorphism and the associated degree and patterns of sex‐specific morphological variation, and therefore have the potential to confound attempts to generate reliable alpha taxonomic hypotheses.

## 3 How Might Variation Associated with Sexual Dimorphism Confound Alpha Taxonomic Assessments Among Extinct Hominin Taxa?

Primate species vary in the degree of sexual size dimorphism, such that in some taxa, adult males can be over twice as heavy as females (e.g., [29, 62, 63, 94]). If we assume that similar levels of sexual size dimorphism may occur among extinct hominin taxa [23], there is the potential for researchers to either overestimate or underestimate the number of species present within a fossil assemblage. A scenario in which the variation within a fossil assemblage exceeds the maximum amount of variation observed among living primate taxa may lead researchers to overestimate the number of species present in a site sample [1, 2, 3] (Box 1).

Box 1: **Two sexes, or two species?**

One of the challenges facing researchers engaged in generating hypotheses about the alpha taxonomy of early hominins is how to assess the significance of variation in fossil evidence that is interpreted as sampling a single species. As sample size increases, so may the range of variation, until the limit present in the species under question has been reached. But at what stage, and under what circumstances, does additional size and shape variation demand that the single‐species hypothesis should be reconsidered, and potentially rejected? We use the example of variation in *Paranthropus boisei* to illustrate these challenges.

The skull and dental morphology of *P. boisei* is so distinctive that for most researchers there is little discussion about which cranial specimens should be allocated to that taxon. Debates about its taxonomy center not on identification, but on how to explain the considerable synchronic variation within the current *P. boisei* craniodental hypodigm (e.g., [12, 104].). Is that variation the result of intra‐specific variation, including substantial sexual dimorphism, within a single species, or is it because the fossil record of multiple species has mistakenly been lumped into the hypodigm of a single species?

Some researchers have concluded that the different patterns of ectocranial cresting, and the differences in the degree of prognathism of the lower face between two large, presumably male crania, OH 5 and KNM‐ER 406, are best interpreted as evidence of intra‐ and not inter‐specific variation [105, 106]. More recent additions to the hypodigm (e.g., two partial crania from Koobi Fora, KNM‐ER 13750 and 23000, and the skull from Konso, KGA 10−525), are also consistent with the observation that while some aspects of cranial morphology, such as the distribution of the ectocranial crests and the shape of the face, vary among members of the presumed male morph of *P. boisei*, other regions (e.g., the cranial base) are relatively invariant [2, 107].

The KNM‐ER 407 calvaria, and the KNM‐ER 732 partial hemicranium, differ in both size and shape from presumed male *P. boisei* crania such as OH 5 and KNM‐ER 406. The morphological differences between KNM‐ER 406 and 407 were so great that the initial taxonomic assessment of the latter placed it in “…either a gracile species of *Australopithecus* or else a very early representative of *Homo*…” [108, p. 224]. In contrast, the initial taxonomic assessment of KNM‐ER 732 suggested that “it seems likely that the two specimens (KNM‐ER 406 and 732) represent the two sexes of the same species” [109, p. 244]. The microstructure of the exposed enamel of the only preserved tooth crown of KNM‐ER 732 is *P. boisei*‐like [110], and the relative size of the postcanine dentition as judged from the proportions of the alveolar process, is within, or just below, the range for *P. boisei* [105]. In addition, the arrangement of the cranial base of KNM‐ER 732 and KNM‐ER 407, especially the relatively anterior position of the foramen magnum and the coronally‐orientated petrous temporal bones, resembles the arrangement seen in OH 5 and KNM‐ER 406 [105, 111]. Erosion has damaged the frontal and zygomatic regions of KNM‐WT 17400 [112], but the remaining osseous and dental morphology leaves little doubt that this subadult specimen belongs to *P. boisei*. Because the *P. boisei* hypodigm at the time comprised crania (e.g., OH 5 and KNM‐ER 406) whose ectocranial morphology was analogous to that of large male members of *Gorilla* and *Pongo*, and because both KNM‐ER 407 and 732 possessed morphological features that, despite their smaller size, were regarded as diagnostic of *P. boisei*, many researchers subscribed to the view that crania such as KNM‐ER 407 and 732 were smaller‐bodied, presumably female, representatives of *P. boisei*, thereby providing evidence of sexual dimorphism within that taxon (e.g., [8, 14, 106, 113, 114]).

It has been suggested that the exceptional degree of size variation in the equivalent mandibular hypodigm may be because it samples more than one hominin taxon [115, 116]. However, much of this “excessive” variation can potentially be explained by changes to these specimens after the death of the individuals. In some specimens, *post mortem* matrix‐filled cracks have artificially inflated the size of the mandibular corpus, whereas in others erosion of surface bone of the corpus has reduced corpus size [105, 117]. When the mandibles of *P. boisei* were assessed for matrix‐filled cracking, or for signs of surface erosion, the incidence of the former was greater in the larger specimens, whereas erosion was more common in the absolutely smaller corpora [117]. Apart from these extrinsic causes for differences in overall size, the size and shape of the mandibular corpus of *P. boisei* is remarkably stable through geological time [2, p. 129, Fig. 2E], and both small and large mandibles in the sample display a characteristically robust (i.e., relatively broad for its height) corpus and rounded base [2, 105]. In addition, Wood et al. [105] demonstrated that the pattern of intra‐specific cranial variation within *P. boisei* was similar to that seen in closely‐related extant taxa. Wood and Lieberman [12] suggested the pattern of cranial regional variability (the tendency of regions within the cranium to vary) was consistent with the hypothesis that regions subjected to high levels of masticatory‐related strain, such as the face and mandible, tend to vary more than in regions such as the cranial base and cranial vault that are subjected to lower levels of strain, but not all researchers agree [118, 119].

There is no compelling evidence to support the hypothesis that the hypodigm of *P. boisei sensu stricto* samples more than one taxon. While *P. boisei sensu stricto* subsumes substantial cranial variation, it is consistent with the hypothesis that high levels of sexual dimorphism in that taxon result in substantial size and shape differences between larger, presumed male, crania, and smaller, presumed female, crania. The intriguing exception is the lack of any substantial dimorphism in the size and shape of the canine crowns and the length of the roots.

**Figure 1:**
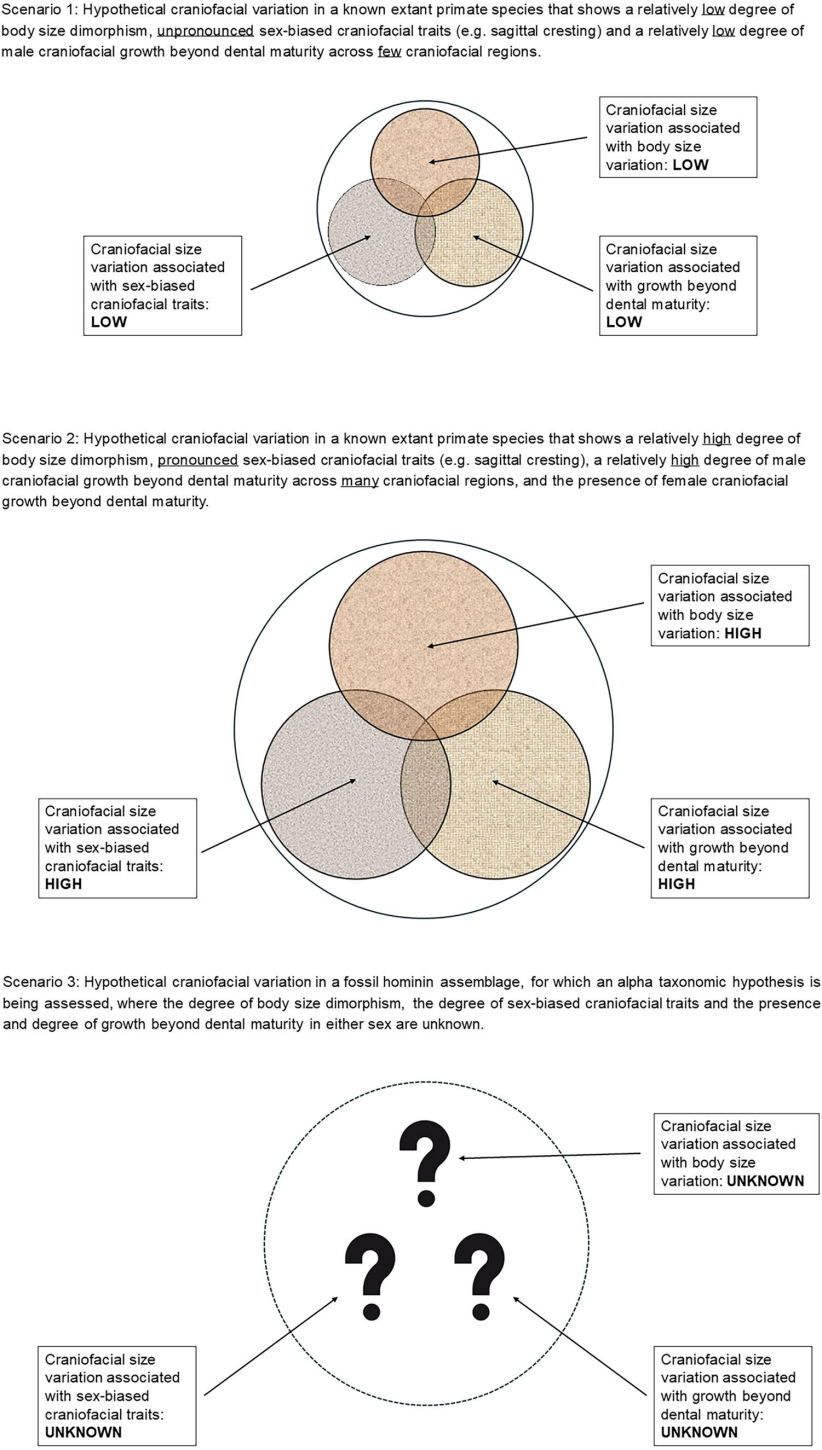
Three hypothetical scenarios, depicting how sexual dimorphism may contribute to the variation within a craniofacial assemblage of an extant species whose alpha taxonomy is well‐supported by various lines of evidence (Scenarios 1 and 2) and for an unknown fossil assemblage for which an alpha taxonomic hypothesis is being generated (Scenario 3). The outer circle in each scenario represents overall sample variability, and the inner circles in Scenarios 1 and 2 correspond to different sources of that variability, which are not independent of one another. The size of each circle represents the magnitude of variation, and the white sections within each circle boundary (Scenarios 1 and 2) represents intra‐specific craniofacial variation not related to sexual dimorphism. In Scenario 3, the alpha taxonomic hypothesis is difficult to assess because the influences of sexual dimorphism, and the nature of any inter‐relationship, are unknown. These hypothetical scenarios focus on the ways sexual dimorphism may contribute to variation within a given assemblage. In reality, influences of taphonomy and geographical and temporal variation, plus functional, biomechanical and other adaptative factors (not depicted here and which are outside the scope of this paper) may also contribute to the observed craniofacial variation. The solid outer circle in Scenarios 1 and 2 reflects the greater reliability of alpha taxonomic hypotheses generated for extant taxa, and the dotted outer circle in Scenario 3 reflects the markedly reduced reliability of the alpha taxonomic hypothesis generated for a fossil hominin assemblage.

We also need to take into account the possibility that, in addition to body size dimorphism contributing to craniofacial variation in a fossil assemblage, sexual selection may be acting on the craniofacial morphology of early hominin taxa in the context of visual signaling [35, 36, 95], or in association with craniofacial growth beyond dental maturity (e.g., [78, 79]), where selection may act on either sex (Section 2; Figure 1). Hominins are characterized by having reduced canine crowns [15], but in non‐human primates canines are involved in social signaling [83, 85, 96]. While the reasons underlying canine crown height reduction in hominins are unclear, some researchers have suggested that the loss of the canine/P3 honing complex is indicative of a reduced scope of sexual selection to target large canine size, or is associated with craniodental adaptations associated with masticatory changes [97, 98, 99, 100, 101]. Whatever the reason for canine size reduction in hominins, in the absence of canines being involved in social signaling, some researchers have suggested that other craniofacial regions may have replaced canine crown height as a means of social signaling among extinct hominin taxa [95, 102]. Regions that are hypothesized to be involved in social signaling are the browridges [10, 35, 37, 103], the mid‐face [41, 43, 95, 102], and the sagittal crest [9]. If a particular morphology is typically only expressed in one sex (e.g., sagittal crest), or if a morphological region is under sexual selection, it would be unwise to interpret variation in those structures and/or regions as evidence of taxic diversity.

## 4 How Might Researchers Assess Which Skeletal Regions to Use to Generate Reliable Alpha Taxonomic Hypotheses?

There is still much to be understood about the extent to which regions of the skeleton vary among modern humans and extant non‐human primates in response to sexual or social selection. This understanding is important for deciding whether evidence from particular morphological regions should be avoided when generating alpha taxonomic hypotheses. High levels of sexual dimorphism, and associated high levels of intra‐specific variation, render these regions especially unreliable for generating alpha taxonomic hypotheses. As noted in Section 2.2, the craniofacial regions that are hypothesized to vary in response to social signaling among primates include browridges [10, 35, 36, 37, 103], the mid‐face [41, 43, 45, 95, 102], and sagittal crest expression and size [9, 74]. These morphological regions have the potential for unique patterns of sexual dimorphism to be expressed within species as a result of sexual or social selection. Further research is required to understand which morphological regions show high intra‐specific variability, potentially associated with sexual or social selection. Once a comprehensive extant comparative framework is established, researchers will be better equipped to understand the extent to which sexual or social selection influences male and female skeletal morphology. Regions hypothesized to vary in response to sexual or social selection should be avoided when generating alpha taxonomic hypotheses.

Closely‐related taxa show similar degrees and patterns of sexual size dimorphism based on phylogenetic relatedness [23, 120], so it is likely the morphological regions showing high levels of sexual dimorphism in closely‐related extant taxa will also do so within an early hominin fossil assemblage. Therefore, alpha taxonomic hypotheses should focus on morphological regions for which there is either no, or weak, evidence that they are under social or sexual selection.

## 5 How Much Information Are We Missing By Focusing Exclusively on Hard Tissue Sexual Dimorphism?

The reality that fossilized hard tissues are all that remains of our early hominin relatives means that we lack an understanding of sexual dimorphism associated with the soft tissues that are commonly sexually dimorphic in extant primates (e.g., the red nasal strip observed in male mandrills, sexual dichromatism in gibbons, the presence of a beard in modern human males, facial flanges in orangutan males, and the enlarged bulbous nose in male proboscis monkeys) (as reviewed in [33, 121]). While there is no direct evidence of these structures, there may be indirect evidence in the form of any hard tissue correlates of these soft tissue features. For example, jaw‐muscle fiber length and architecture is associated with jaw size variables and maximum jaw gape among extant cercopithecoid males, associated with canine displays and aggressive biting [122, 123]. Any evidence of a link between sagittal crest size and fat hump size in extant gorillas, or facial muscle markings and facial flanges in extant orangutans, would enable researchers to use hard tissue evidence in the hominin fossil record to reconstruct soft tissue morphology, and thus infer sex. In the absence of being able to obtain DNA data from early hominin remains, which has been possible for some later hominin species [124, 125, 126], the ability to reconstruct sexual dimorphism in facial markings, body hair or skin color from the fossil record is limited. However, developments in techniques for extracting the components of amelogenin proteins from fossilized remains have opened up the prospect that researchers will be able to determine sex by using peptides recovered from individual fossil specimens ([127, 128], submitted).

## 6 Conclusion

If, as is the case in extant primates, parts of the skeleton and dentition of extinct hominins were influenced by sexual or social selection, evidence from these regions should be avoided when generating alpha taxonomic hypotheses. A more comprehensive understanding of the biological basis of observed patterns of sexual dimorphism among extant primates will help us better understand how sexual dimorphism contributes to intra‐specific variation within early hominin taxa. The limitation of fossilized hard tissues being the sole source of evidence means we lack an understanding of important aspects of sexual dimorphism in early hominins. Research that investigates whether there are hard tissue correlates of soft tissue dimorphism has the potential to reduce any confounding effects of sexual dimorphism.

## Conflicts of Interest

The authors declare no conflicts of interest.

## Data Availability

No new data were generated in this study, therefore data availability is not applicable to this article.
